# Cell-type specific epigenetic and transcriptional mechanisms in substance use disorder

**DOI:** 10.3389/fncel.2025.1552032

**Published:** 2025-03-28

**Authors:** Bin Wang, Jiale Wang, Nicholas J. Beacher, Da-Ting Lin, Yan Zhang

**Affiliations:** ^1^National Institute on Drug Dependence and Beijing Key Laboratory of Drug Dependence Research, Peking University, Beijing, China; ^2^School of Basic Medical Sciences, Peking University Health Science Center, Beijing, China; ^3^Intramural Research Program, National Institute on Drug Abuse, Baltimore, MD, United States; ^4^The Solomon H. Snyder Department of Neuroscience, Johns Hopkins University School of Medicine, Baltimore, MD, United States

**Keywords:** substance use disorder, epigenetic regulation, transcriptional adaptations, cell-type specific modifications, neuroadaptive mechanisms

## Abstract

Substance use disorder (SUD) is a chronic and relapse-prone neuropsychiatric disease characterized by impaired brain circuitry within multiple cell types and neural circuits. Recent advancements in single-cell transcriptomics, epigenetics, and neural circuit research have unveiled molecular and cellular alterations associated with SUD. These studies have provided valuable insights into the transcriptional and epigenetic regulation of neuronal and non-neuronal cells, particularly in the context of drug exposure. Critical factors influencing the susceptibility of individuals to SUD include the regulation of gene expression during early developmental stages, neuroadaptive responses to psychoactive substances, and gene–environment interactions. Here we briefly review some of these mechanisms underlying SUD, with an emphasis on their crucial roles in in neural plasticity and maintenance of addiction and relapse in neuronal and non-neuronal cell-types. We foresee the possibility of integrating multi-omics technologies to devise targeted and personalized therapeutic strategies aimed at both the prevention and treatment of SUD. By utilizing these advanced methodologies, we can gain a deeper understanding of the fundamental biology of SUD, paving the way for more effective interventions.

## 1 Introduction

Substance use disorder (SUD) is a complex and highly predisposing neuropsychiatric disease, characterized by excessive drug use, tolerance, and chronic relapse (Koob and Volkow, 2010). SUD is associated with persistent and recurrent disturbances within multiple brain circuits, for example, the mesolimbic dopamine system (Koob, 1992). Exposure to drugs of abuse changes transcriptional and epigenetic regulation of neuronal and non-neuronal (e.g., Microglia) cells (Nogueira et al., 2019; Facciol et al., 2020; Bestry et al., 2024; Gonzalez-Flores et al., 2024). These are further perturbed and damaged during the development of chronic SUD and are linked to differences in predisposition to develop SUD as defined below. Here, we review transcriptional and epigenetic adaptations in different cell types in SUD in animal models and humans. We discuss how adaptations occur during different phases of SUD and are linked to individual differences in how early developmental gene expression regulation and gene–environment interactions contribute to SUD susceptibility. Finally, we present a perspective on the potential future clinical applications and therapeutic potentials.

## 2 Transcriptional, genetic, and epigenetic associations with SUD

Substance use disorder is a multifactorial disease influenced by a combination of genetic and environmental factors. Transcriptional mechanisms act to create messenger RNA (mRNA) from DNA which are then translated into proteins in cells (Dynan and Tjian, 1985). Transcription factors (TF) are proteins which regulate the expression of specific genes (Yusuf et al., 2012), but can be altered resulting from SUD. ΔFosB, for example, is a TF infamously described as a ‘molecular switch’ in SUD by overexpressing in the brain’s reward system (Nestler et al., 2001). ΔFosB is associated with drug craving and an elevated risk of relapse (Winstanley et al., 2007) and is associated with long term plastic adaptations of reward circuits which exacerbate addictive behaviors (Gajewski et al., 2019). SUD alters expression of hundreds of genes, leading to maladaptive changes at the molecular, cellular, and circuit levels, which in turn promote drug seeking behaviors (Walker et al., 2018). Early exposure to addictive substances enhances the sensitivity of reward system in the brain (Caster and Kuhn, 2009). Dopaminergic neurons exhibit distinct transcriptional responses to drug exposure during childhood and adolescence compared to adulthood, which are crucial in the development of drug dependence (Caster and Kuhn, 2009). Environmental stressors and drug exposure act synergistically to enhance the reward response to addictive substances by altering the transcriptional responses of neurons involved in stress regulation, intensifying drug cravings and dependence (Jiang et al., 2023).

Epigenetics is defined by a broad range of molecular mechanisms that regulate gene expression without altering the actual DNA sequence itself (Allis and Jenuwein, 2016; Sartor, 2019). This includes factors from the environment and individual behaviors that can alter chromatin dynamics and capabilities of DNA transcription (Conaway, 2012), which includes DNA methylation and post-translational modifications of histones (such as acetylation, methylation, and phosphorylation) (Allis and Jenuwein, 2016; Farrelly et al., 2019; Sartor, 2019). In the nervous system, DNA methylation and histone modifications are of critical importance with regard to the development and maintenance of neuronal networks, learning and memory processes, and behavioral responses to environmental stimuli (Conaway, 2012). Studies have implicated epigenetic alterations linked to persistent pathophysiological changes associated with neuronal function and disease susceptibility (Houston et al., 2013). Chronic exposure to drugs of abuse induces long-term epigenetic modifications in the reward circuit in the brain (Cheron et al., 2023), which in turn give rise to enduring functional alterations in neural circuits (Kaplan et al., 2022; Koijam et al., 2024). These modifications play a critical role in reinforcing drug-related behaviors and promote drug dependence (Hamilton and Nestler, 2019). Epigenetic adaptations play a pivotal role not only in the progression of SUD but also in determining an individual’s susceptibility to addiction. For example, gene expression regulation during early developmental stages, such as DNA methylation and histone modifications, could persist into adulthood, making individuals more susceptible to drug addiction (Browne et al., 2020). After drug exposure, epigenetic marks may leave persistent traces in the transcriptional regulation of individual neurons and influence physiological responses in subsequent stages (Nogueira et al., 2019). For example, the upregulation of certain transcription factors (such as FosB) and neurotransmitter receptor genes closely associated with mesolimbic reward plasticity changes after drug exposure (Nestler et al., 2001; Winstanley et al., 2007; Grueter et al., 2013; Robison and Nestler, 2022). These findings support theories of transgenerational ‘heritability’ of SUD (Deak and Johnson, 2021) and suggest it is both genetic and epigenetic factors that influence individual predisposition to develop SUD (Winstanley et al., 2007; Walker et al., 2018; Hamilton and Nestler, 2019).

## 3 Neurons

The mesolimbic dopamine system functions to promote reward seeking in naturalistic settings, for example, for food (Parada et al., 1990; Carelli et al., 2000; Wise, 2006; Nicola, 2016), water (Parada et al., 1990; Carelli et al., 2000), and sexual reward (Fibiger, 1993; Hull et al., 2004). Drugs of abuse are thought to overwhelm these processes, and cause long term plastic adaptations in these same reward-associated brain regions (Self, 2004; Koob, 2008; Tan et al., 2024). Consequently, this results in drugs hijacking these systems and obsession with drugs despite negative associations and consequences (Volkow, 2005; Koob and Volkow, 2010; Koob, 2013b; Pickard and Ahmed, 2016). The onset and progression of SUD involves dysregulation across multiple neurotransmitter systems, which are closely linked to transcriptional and epigenetic changes in various neural cell types (Hamilton and Nestler, 2019; Nestler and Lüscher, 2019; Anderson and Taniguchi, 2022; Koijam et al., 2024). Exposure to addictive drugs leads to the transcriptional adaptations of key genes such as Brain-Derived Neurotrophic Factor (BDNF) and Activity-Regulated Cytoskeletal-associated Protein (ARC), which directly influence the synaptic strength and neural plasticity (Huggett and Stallings, 2020; Chen et al., 2024). We describe a sample of these epigenetic and transcriptional adaptations in crucial brain regions and cell-type specific changes of neurons unique to those systems.

### 3.1 Nucleus accumbens

Motivated movements are theorized to arise by way of spiraling ascending limbic projections (Haber et al., 2000). These projection systems are thought to be ‘gated’ by deep brain neurons such as those in the nucleus accumbens (NAc) (Morrison et al., 2017). NAc receives input from a multitude of regions associated with SUD (Britt et al., 2012) and are predominantly made of inhibitory GABAergic medium spiny neurons (MSNs) (Gerfen et al., 1990) thought to disinhibit downstream projections to premotor and motor cortices and guide motivated movements (Morrison et al., 2017). Drugs of abuse can alter this dynamic and cause changes to this aspect of the projection system (MacAskill et al., 2014). This results in situations where NAc neurons signal drug availability even during ‘drug-free’ states (Estrin et al., 2023). Individuals are thought to maintain a ‘preferred’ micromolar brain level of certain drugs (e.g., cocaine) by self-administering in predictable patterns to achieve a desired ‘satiety’ state (Zimmer et al., 2013). This floods the brain with drug and induces changes to the transcriptional, and epigenetic mechanisms regulating these cells (Huggett and Stallings, 2020; Chen et al., 2024).

Drug use induces a wide range of changes to the NAc transcriptional mechanisms (Nestler and Lüscher, 2019; Mews et al., 2024) including CREB (Carlezon et al., 1998; Dong et al., 2006) and FosB (Grueter et al., 2013). Epigenetic mechanisms in NAc are also disrupted during drug use (Anderson and Taniguchi, 2022). For example, DNA methylation is typically induced by DNA methyltransferases (DNMTs) (Bestor, 2000). Administration of cocaine increases DNMT3A and DNMT3B induced hypermethylation in NAc (Anier et al., 2010) and enhances behavioral sensitization but is reversed by DNMT inhibition (Anier et al., 2010). This coincides with transcriptional downregulation of protein phosphatase-1 catalytic (PP1c) promoter and upregulation of NAcΔFosB (Anier et al., 2010). Further, histone modifications in NAc (Anderson et al., 2019; Werner et al., 2021) are involved in drug-induced NAc plasticity (Maze et al., 2010; Damez-Werno et al., 2016) and are associated with heightened risk of drug relapse (Walker et al., 2015). Long-term drug use also alters NAc chromatin structure (Mews et al., 2024). MSNs exist subtypes D1 and D2 (Gerfen et al., 1990; Lobo and Nestler, 2011). Inhibition of transcription of DMNT3A2 in NAc-D1 MSNs inhibits cocaine seeking reduces inductions of genes such as Arc, FosB, and Egr2 (Bisagno and Cadet, 2019; Koijam et al., 2024). Suppression of histone methyltransferase G9a of D1-MSNs results in reduced cocaine-seeking, while D2 G9a suppression enhances cocaine seeking (Maze et al., 2014; Peña et al., 2014). Histone deacetylase-3 (HDAC3) is hyper-expressed in NAc-D1 MSNs following cocaine exposure and acts to promote cocaine seeking (Campbell et al., 2021). Conversely, downregulation of histone methylation in D2-MSNs is thought to act as a ‘break’ for development of addiction (Damez-Werno et al., 2016). Removal of BDNF receptor TrkB in D2-MSN reduces cocaine-seeking while the opposite holds true for NAc D1-MSNs (Lobo et al., 2010). Opposing D1 and D2 findings are also present regarding egr3 (a cocaine-associated gene) and cocaine-seeking behaviors (Chandra et al., 2017). These changes and adaptations are well documented and suggest drug use of any kind disturbs ‘typical’ functioning of genetic and epigenetic regulation in NAc MSNs.

### 3.2 Basolateral amygdala

The basolateral amygdala (BLA) is thought to encode a host of cognitive processes including fear (Rogan et al., 1997; Gale et al., 2004), affect (Grace and Rosenkranz, 2002), and reinforcement (Gale et al., 2004; Tye et al., 2008; Namburi et al., 2015). The BLA is also associated with motivation (Floresco and Ghods-Sharifi, 2007; Grabenhorst et al., 2012), drug-seeking (Everitt and Wolf, 2002; See, 2002; Stefanik and Kalivas, 2013), and drug craving (Bonson et al., 2002). Neurons in BLA respond to cues predictive of reward (Sugase-Miyamoto and Richmond, 2005). Excitatory glutamatergic principle output neurons are the predominant NAc-projecting neurons in the BLA (McDonald, 1992; Grace and Rosenkranz, 2002; Russo and Nestler, 2013) while BLA inhibitory GABAergic neurons are thought to modulate the activity of glutamatergic neurons (Ahmed and Paré, 2023). It is thought that BLA projects to NAc and PFC via excitatory projections (Shinonaga et al., 1994; Ambroggi et al., 2008; Stuber et al., 2011; Britt et al., 2012; Janak and Tye, 2015) theorized to be necessary for “cue-induced” reward seeking (Di Ciano and Everitt, 2004; Ambroggi et al., 2008; Shiflett and Balleine, 2010). Epigenetic influences of BLA have been shown *via* inhibition of DNMT reduces cue-induced heroin seeking (Qian et al., 2022), while inhibition of histone deacetylase (HDAC) enhances morphine-seeking and enhances activation of BDNF, ΔFosB, and CREB (Wang et al., 2015). In general, BLA gene expression is ‘globally’ altered in humans suffering from alcoholism resulting from histone modification and DNA hypomethylation (Ponomarev et al., 2012). Further, heightened BLA c-FOS and mRNA changes associated with pyramidal cells have been modeled in rats exposed to alcohol during adolescence (Vázquez-Ágredos et al., 2024).

### 3.3 Prefrontal cortex

The prefrontal cortex (PFC) is associated with drug seeking behavior (Glanzberg et al., 2024). The PFC is a crucial component involved in feed-forward limbic processing. For instance, PFC receives BLA signals (Burgos-Robles et al., 2017; Crofton et al., 2022) and transmits excitatory projections with BLA and NAc (Ambroggi et al., 2008; Stuber et al., 2011; Otis et al., 2017; Li et al., 2023) during reward seeking. The neural circuit dynamics between these critical brain regions have been demonstrated to facilitate motivated responding (Ambroggi et al., 2008; Ghazizadeh et al., 2012; Tomasi and Volkow, 2013; Thomas et al., 2020). Early-life cocaine exposure alters a host of PFC gene expression that appeared as early as day 1 (e.g., Egr1/2, and Jmjd1a), while some genes (and histone methylation) endured into adulthood (Black et al., 2006). Chronic cocaine addiction promotes PFC DNA methylation at BDNF promoter regions in humans (Poisel et al., 2023), and histone acetylation in rat DNA increases BDNF transcription in the PFC (Sadri-Vakili et al., 2010). Morphine withdrawal is associated with epigenetic regulation of BDNF *via* chromatin remodeling (Wang et al., 2012). Early alcohol exposure increases histone acetylation at the PFC BDNF promotor and transcription sites (Montesinos et al., 2016). Neurons in the PFC include the excitatory projection pyramidal cells (Krimer and Goldman-Rakic, 2001) and the inhibitory interneurons that act to modulate the local activity of pyramidal cell signaling (Pouille and Scanziani, 2001; Isaacson and Scanziani, 2011; Hu et al., 2014; Ferguson and Gao, 2018). Morphine administration disinhibits these inhibitory neurons and enhances reward (Jiang et al., 2021). Interestingly, direct infusion of BDNF into PFC reduces drug seeking by modulating pyramidal glutamatergic signals to NAc (Berglind et al., 2009; Whitfield et al., 2011). Supporting this notion, ‘knockdown’ of PFC BDNF amplifies cocaine seeking *via* the same glutamatergic process (Sadri-Vakili et al., 2010). Heroin use in humans is associated with altered DNA methylation and glutamatergic plasticity in the PFC (Kozlenkov et al., 2017). Finally, human genome-wide ‘addiction-risk’ assessments found chromatin modulations and enhanced susceptibility for initiation of cannabis use and cigarette smoking among excitatory, but not inhibitory, neurons in PFC (Kozlenkov et al., 2017; Barker and Hines, 2020; Huggett and Stallings, 2020; Sundar et al., 2021; Chen et al., 2024).

## 4 Glial cells

In addition to neurons, glia is an additional subtype of cells involved in typical mammalian neurophysiology. In the central nervous system, major glia cell-types typically include astrocytes, microglia, and oligodendrocytes (Silver et al., 2015). They are essential for a host of critical supportive neurological processes and are impacted by drugs of abuse. We present a brief summary of epigenetic and transcriptional changes in glia below.

### 4.1 Astrocytes in SUD

Astrocytes maintain homeostasis in the brain by maintaining the blood brain barrier, regulating synaptic transmission, neurotransmitter concentrations, energy metabolism (Lee et al., 2022). Following drug exposure, astrocytes exhibit significant transcriptional and epigenetic adaptations that affect the plasticity and function of neural circuits, which highlights the complexity and multifaceted nature of SUD (Bachtell et al., 2017). When exposed to pharmacological agents, astrocytes undergo transcriptional adaptations primarily related to glutamate transport, energy metabolism, and inflammatory responses (Holt and Nestler, 2024). Prolonged exposure to pharmacological agents reduces in the expression of the glutamate transporter (GLT-1, also known as EAAT2), leading to the elevated extracellular glutamate concentrations, which induce neurotoxicity and disrupt synaptic plasticity (Kruyer and Kalivas, 2021). Astrocytes support neuronal energy demands through the lactate-glucose-glutamate metabolic cycle (Petit and Magistretti, 2015). Drug exposure may exert an influence on this process through transcriptional regulation (Mahna et al., 2018). For instance, following alcohol exposure, the expression of monocarboxylate transporters (MCT1 and MCT2) in astrocytes is downregulated, which results in an imbalance in lactate metabolism and may relate to the development of drug dependence (Tan et al., 2022).

### 4.2 Microglia and inflammation in SUD

Microglia are seen as the principal immune cells in the central nervous system (Aloisi, 2001). Microglia maintain brain homeostasis by clearing damaged tissue, releasing inflammatory factors in response to injury and infection, and by facilitating neural circuit repair (Aloisi, 2001; Borst et al., 2021). However, chronic drug exposure induces excessive microglia activation (Li et al., 2024). This is theorized to lead to a reinforcing inflammatory state characterized by release of inflammatory factors and recruitment of additional immune cells (i.e., chronic inflammation) (Loftis et al., 2011; Chan et al., 2015; Piepenbrink et al., 2016; Prakash et al., 2017) associated with sickness behavior which mirrors human reports of depression (Dantzer et al., 2008). People suffering from SUD report high rates of depression (Koob, 2013a; Sullivan, 2018) and drugs of abuse temporarily suppress the activation of certain pro-inflammatory cytokines (Bayer et al., 1995; Baldwin et al., 1998), theoretically providing short-term relief. However, these result in long-term hyperactive responses to sickness (Buchanan et al., 2010; Loftis et al., 2011) and immune activation in response to drug withdrawal (Wang et al., 2021). Microglial activation is frequently accompanied by a substantial upregulation of the NFκB signaling pathway, which regulates the expression of a number of genes involved in the inflammatory response, including IL-6 and TNF-*α* (Jones, 2020). In chronic opioid exposure models, an elevated expression of IL-6 in the microglia has been strongly correlated with the disruption of synaptic plasticity in the nucleus accumbens, which may represent an important molecular mechanism underlying drug craving (Guo et al., 2023). Therefore, it is likely that inflammation changes neuronal communication which leads to changes in behavior and mood. These changes can make it difficult for individuals to stop using opioids (Guo et al., 2023) and contributes to negative reinforcing aspects of addiction (Koob, 2013b; Jones, 2020; Guo et al., 2023).

### 4.3 Oligodendrocytes in SUD

Oligodendrocytes are thought to promote myelination of axons in the central nervous system (Stadelmann et al., 2019). Recent research indicates myelin integrity is closely associated with neuronal function in diseases such as multiple sclerosis (Steinman, 1996). Myelin integrity is linked to an array of neurobehavioral dysfunctions and compulsive behavioral disorders (including SUD) (Sequeira and Turecki, 2006; Nave and Ehrenreich, 2014). Binge-eating, for instance, results in an altered transcriptome and downregulation of oligodendrocytes (Kirkpatrick et al., 2017; Babbs et al., 2020) and impacts reward-related behavior (Babbs et al., 2020). Finally, alcoholism is associated with altered transcription factors and histone modifications resulting in gene expression alteration in oligodendrocytes (Miguel-Hidalgo, 2018).

### 4.4 Technological advancements/clinical applications

In recent years, the application of single-cell transcriptomics has provided new opportunities to explore cell-type-specific transcriptional adaptations in substance use disorder (SUD). By conducting high-precision analysis of the transcriptomes of individual cells, researchers are now able to reveal the dynamic changes in different cell populations after drug exposure and identify key genes and molecular pathways associated with addiction progression and susceptibility. Single-cell RNA sequencing (scRNA-seq) enables the differentiation of gene expression changes across heterogeneous cell populations, particularly in complex brain regions such as the nucleus accumbens, hippocampus, and dorsolateral prefrontal cortex, which are important in the pathogenesis of SUD (Tran et al., 2021).

Moreover, epigenetic research has provided new perspectives on the regulatory mechanisms underlying transcriptional adaptations. Drug exposure not only affects gene transcription levels but also influences transcriptional adaptations in specific cell types by altering epigenetic marks, including DNA methylation and histone modifications (Cheron et al., 2023),which adds complexity to our understanding of how drugs reshape the transcriptional landscape of the brain. Future studies can combine epigenetics with single-cell genomics to explore how epigenetic regulation affects cell-type-specific transcriptional adaptations, ultimately influencing the progression of SUD and individual susceptibility (Nohesara et al., 2024). This multidisciplinary approach holds the potential to unveil novel therapeutic targets and personalized intervention strategies for SUD.

A deeper understanding of how specific cell-type transcriptional and epigenetic adaptations to the progression and susceptibility of SUD will provide new therapeutic targets for precision medicine. For instance, by regulating the expression of addiction-related transcription factors (e.g., ΔFosB) and targeting epigenetic modifications in non-neuronal cells may facilitate the development of more precise drugs to reverse the molecular changes induced by drug exposure (Robison and Nestler, 2022). Additionally, treatment strategies based on non-coding RNAs (e.g., miRNAs) and the application of CRISPR technology has opened new avenues for the personalized treatment of addictive disorders (Tan et al., 2024).

Moving forward, multi-omics and cutting-edge molecular techniques to deeply probe will offer new insights into dynamic alterations in various cell types across the addiction cycle. It will pave the way for the development of more targeted strategies for precise intervention and individualized treatment of SUD.

## 5 Conclusion

In summary, SUD is a multifaceted neuropsychiatric disease resulting from complex interactions between genetic and epigenetic factors in neuronal and non-neuronal cells. The transcriptional and epigenetic adaptations in both neuronal and non-neuronal cells play a crucial role in the onset, progression, and relapse of SUD. Recent advancements in single-cell transcriptomics and epigenetics have provided valuable insights into the cell-type-specific molecular changes underlying these processes. Understanding the early developmental gene expression regulation, drug-induced neuroadaptive responses, and gene–environment interactions is essential for identifying new biomarkers and therapeutic targets. By integrating multi-omics technologies and advanced molecular tools, future research will lead to more effective, individualized approaches for preventing and treating SUD, paving the way for improved clinical outcomes.
